# Defensive Perimeter in the Central Nervous System: Predominance of Astrocytes and Astrogliosis during Recovery from Varicella-Zoster Virus Encephalitis

**DOI:** 10.1128/JVI.02389-15

**Published:** 2015-12-17

**Authors:** John E. Carpenter, Amy C. Clayton, Kevin C. Halling, Daniel J. Bonthius, Erin M. Buckingham, Wallen Jackson, Steven M. Dotzler, J. Patrick Card, Lynn W. Enquist, Charles Grose

**Affiliations:** a Divisions of Virology and Neurology, Children's Hospital, University of Iowa, Iowa City, Iowa, USA; b Department of Laboratory Medicine and Pathology, Mayo Clinic, Rochester, Minnesota, USA; c Department of Neuroscience, University of Pittsburgh, Pittsburgh, Pennsylvania, USA; d Department of Molecular Biology, Princeton University, Princeton, New Jersey, USA

## Abstract

Varicella-zoster virus (VZV) is a highly neurotropic virus that can cause infections in both the peripheral nervous system and the central nervous system. Several studies of VZV reactivation in the peripheral nervous system (herpes zoster) have been published, while exceedingly few investigations have been carried out in a human brain. Notably, there is no animal model for VZV infection of the central nervous system. In this report, we characterized the cellular environment in the temporal lobe of a human subject who recovered from focal VZV encephalitis. The approach included not only VZV DNA/RNA analyses but also a delineation of infected cell types (neurons, microglia, oligodendrocytes, and astrocytes). The average VZV genome copy number per cell was 5. Several VZV regulatory and structural gene transcripts and products were detected. When colocalization studies were performed to determine which cell types harbored the viral proteins, the majority of infected cells were astrocytes, including aggregates of astrocytes. Evidence of syncytium formation within the aggregates included the continuity of cytoplasm positive for the VZV glycoprotein H (gH) fusion-complex protein within a cellular profile with as many as 80 distinct nuclei. As with other causes of brain injury, these results suggested that astrocytes likely formed a defensive perimeter around foci of VZV infection (astrogliosis). Because of the rarity of brain samples from living humans with VZV encephalitis, we compared our VZV results with those found in a rat encephalitis model following infection with the closely related pseudorabies virus and observed similar perimeters of gliosis.

**IMPORTANCE** Investigations of VZV-infected human brain from living immunocompetent human subjects are exceedingly rare. Therefore, much of our knowledge of VZV neuropathogenesis is gained from studies of VZV-infected brains obtained at autopsy from immunocompromised patients. These are not optimal samples with which to investigate a response by a human host to VZV infection. In this report, we examined both flash-frozen and paraffin-embedded formalin-fixed brain tissue of an otherwise healthy young male with focal VZV encephalitis, most likely acquired from VZV reactivation in the trigeminal ganglion. Of note, the cellular response to VZV infection mimicked the response to other causes of trauma to the brain, namely, an ingress of astrocytes and astrogliosis around an infectious focus. Many of the astrocytes themselves were infected; astrocytes aggregated in clusters. We postulate that astrogliosis represents a successful defense mechanism by an immunocompetent human host to eliminate VZV reactivation within neurons.

## INTRODUCTION

Varicella-zoster virus (VZV) is an alphaherpesvirus that can cause infections in both the peripheral nervous system (PNS) and the central nervous system (CNS). The neurotropism of VZV is manifested most commonly in the PNS as herpes zoster (shingles) (1). Herpes zoster is the reactivation of the same virus that entered a latent state decades earlier during primary VZV infection. The most common sites of VZV latency are the dorsal root ganglia and the trigeminal ganglia (2). Herpes zoster has been investigated in both human samples and in the severe combined immunodeficient mouse/dorsal root ganglion model (3–5).

Upon reactivation from the trigeminal ganglion, herpes zoster ophthalmicus is the prototypical expression of trigeminal zoster. In addition to innervation of the skin of the upper face, sensory afferent fibers originating in the V1 and V2 branches of trigeminal ganglion travel into the meninges of the frontal and temporal lobes, respectively (6, 7). Furthermore, VZV reactivation within the trigeminal ganglion does not necessarily lead to virus exit within the same fibers it entered following primary infection. As a result, VZV can travel in afferent fibers from the trigeminal ganglion to the cerebrum, where it can cause CNS disease in the absence of a clinically apparent zoster exanthem.

In contrast to peripheral herpes zoster, samples of brain tissue from a human who recovers from a VZV infection of the CNS are exceedingly rare. We previously published a brief case report of focal VZV encephalitis (8). The lesion (∼1 cm in diameter) was located mainly in the gray matter of the temporal lobe. The infection likely arose in the pia arachnoid overlying the gray matter, innervated by terminal fibers of the meningeal nerve emanating from the V1 branch of the trigeminal ganglion. Because a low-grade glioma was the initial diagnosis, the entire lesion was removed neurosurgically. When a pathology examination failed to confirm the diagnosis of tumor, a preliminary investigation of possible viral pathogens detected VZV antigens and wild-type VZV DNA in the brain tissues.

Following an extensive literature search regarding alphaherpesvirus encephalitis, it was apparent that almost no neuropathology data were available about the cellular response to VZV encephalitis in a human subject who overcame his infection, as opposed to VZV-infected brain samples collected postmortem (9–11). Postmortem brain samples may not provide insight into cells required for recovery from VZV infection; in fact, postmortem samples from immunocompromised subjects likely represent a failure of an as yet poorly characterized response to contain or eliminate a herpesvirus infection. Our subsequent more detailed investigation of the VZV-infected brain tissue found an unexpected predominance of infected astrocytes and astroglioisis. Because there is no animal model for VZV encephalitis by which to compare our findings in this human brain sample, we reexamined older reports of CNS infection with the closely related pseudorabies virus (PRV) (12). Our subsequent PRV studies confirmed a strikingly similar cellular response found in one model of experimental PRV encephalitis.

## MATERIALS AND METHODS

### Reagents for identification of VZV proteins and cell types in brain.

The anti-VZV reagents included the following monoclonal antibodies (MAbs): 3B3 (anti-glycoprotein E [anti-gE]), 233 (anti-gC), 206 (anti-gH), and human anti-gH. All murine MAbs have been characterized (13–15); likewise, the human anti-gH MAb has been described (16). All MAb reagents have been shown not to react nonspecifically with human tissue; since the human subject was not of blood group A, there was no concern about nonviral reactivity of antibody to A antigen in brain cells (8, 17). The anticell reagents included a rabbit antineurofilament antibody against neurons (Sigma N4142), mouse anti-GFAP MAb (Sigma G3893), and cyanine 3(Cy3)-conjugated mouse anti-glial fibrillary acidic protein (anti-GFAP) MAb (Sigma C9205) against astrocytes, mouse antibody against oligodendrocytes (RIP; NIH Developmental Studies Hybridoma Bank), and a red fluorescent Griffonia (Bandeiraea) simplicifolia plant lectin I, isolectin B4 (IB4) (Invitrogen 121412), against microglia. For the reagents not conjugated with a fluoroprobe, we purchased the following secondary reagents: Alexa 488, 546, and 633 fluoroprobes conjugated to a goat anti-rabbit IgG, goat anti-mouse IgG, or goat anti-human IgG (Invitrogen).

### Protocols for confocal microscopy and Imaris software.

The brain biopsy specimen had been flash-frozen at −70C. For confocal microscopy, several 10-μm frozen sections were cut in a cryostat and adhered to glass slides. The sections were incubated with antibody probes by the described methods and then viewed with a Zeiss LSM710 spectral confocal microscope, using 10×, 20×, 40×, and 63× high-numerical-aperture oil immersion objective lenses (15, 18). Image size was set to either 512 by 512 or 1,024 by 1,024 pixels. Emission detection bandwidths were configured by Zeiss Zen control software. The confocal pinhole was set to 1 Airy unit. The z-stack acquisition intervals were selected to satisfy Nyquist sampling criteria. To further assess colocalization of labeled proteins in individual labeled brain cells, three-dimensional (3D) animations were produced from z-stacks comprising up to 40 images, with the aid of Imaris software (Imaris version 7.6 reference manual; Bitplane).

### Protocol for TEM of human tissue.

For electron microscopy, the frozen brain sample was thawed to approximately −10°C, and then a small piece (2 by 2 by 2 mm) was excised. That piece was further subdivided and the sections placed in BEEM capsules containing 2% glutaraldehyde in phosphate-buffered saline (PBS) at 4°C. The samples were then prepared for transmission electron microscopy (TEM) using previously described methodology (19). Briefly, the sample was further fixed in 1% OsO_4_ plus 1.5% KFe(CN)_6_ in PBS for 2 h; washed twice with water, and then further stained with 2% uranyl acetate in water for 30 min. The tissue was then dehydrated in a graded series of ethanol solutions: 25%, 50%, and 75% for 15 min each followed by 100% ethanol twice for 30 min each, 50% ethanol–Epon (Eponate 12) for 2 h, 100% Epon for 2 h, and 100% Epon for 18 h. The tissue was then polymerized with 100% Epon at 70°C for 18 h. Microtomy yielded thin sections of the embedded brain tissue that were then placed on TEM grids, further stained with 4% uranyl acetate in water and then 1% lead citrate with water, washing after each stain. The grids were then air dried and imaged using a JEOL 1230 transmission electron microscope fitted with a Gatan camera.

### Protocol for rat pseudorabies microscopy study.

Two adult male Sprague-Dawley rats weighing approximately 300 g were used in the investigation. The animals were acclimated to the animal housing unit in a biosafety level 2 (BSL-2) laboratory for 2 days prior to the onset of the experiment. The temperature (22 to 25°C) and light-dark cycle (12 h of light, with light on at 0700) were standardized in the colony room, and food and water were available *ad libitum*. On the day of the experiment, the animals were anesthetized with isoflurane, and their heads were secured in a stereotaxic frame. Following shaving of the head and sterilization of the scalp with Betadine, an incision was made to expose the dorsal surface of the skull. A beveled 1-μl Hamilton syringe loaded with PRV strain RV-152 was lowered into the interpositus nucleus through a craniotomy drilled at coordinates obtained from the rat brain atlas of Swanson (20). Virus was injected into the interpositus nucleus at 10 nl/min for a final volume of 100 nl using a nanoliter pump (model 310; Stoelting). Ten minutes following completion of the injection, the needle was removed from the brain, the craniotomy was filled with bone wax, the scalp was closed with wound clips, and the animal was placed under a heat lamp to recover consciousness. When fully ambulatory, it was returned to its home cage. At 48 h following virus injection, each animal was deeply anesthetized with 390 mg/ml sodium pentobarbital (Fatal Plus; Vortech Pharmaceuticals) and perfused transcardially with buffered aldehyde solutions containing 5% glutaraldehyde and 4% paraformaldehyde in 0.1 M sodium cacodylate buffer. The brain was removed, postfixed in the same fixative overnight at 4°C, and sectioned serially at 100 μm/section through the rostrocaudal extent of the inferior olive. Sections were postfixed for 1 h at room temperature in 1% osmium tetroxide and 1.5% potassium ferricyanide in the cacodylate buffer. The sections were washed in multiple changes of buffer, dehydrated in a graded series of ethanols, passed through three changes of acetone, and then infiltrated and flat embedded with Epon-Aradite plastic resin, according to previously published procedures (21). Alternating series of thick and ultrathin sections were cut using a Leica Ultracut E ultramicrotome. Thick sections were stained with toluidine blue. Ultrathin sections were collected on Formvar-coated slot grids, stained with uranyl acetate and lead citrate, and examined and photographed using a Morgagni transmission electron microscope.

(This study was submitted to the Institutional Review Board at the University of Iowa, who deemed the human research exempt. All experimental procedures involving rats conformed to regulations stipulated in the National Research Council's *Guide for the Care and Use of Laboratory Animals* (48) and were approved by the University of Pittsburgh Institutional Animal Care and Use Committee.)

## RESULTS

### VZV transcriptome within the focal encephalitis.

**FIG 1:**
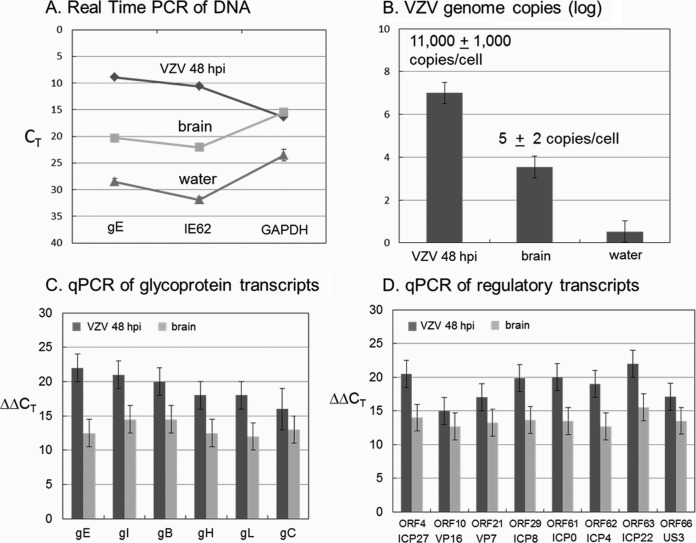
VZV DNA and RNA analyses. DNA and mRNA were extracted from two different pieces of human brain tissue as described in Materials and Methods. (A) VZV genomes. Using DNA from VZV-infected MeWo cells at 48 h postinfection (hpi) (diluted 1:10) as a positive control and DNA from a water sample from the laboratory bench as a negative control, real-time PCR was carried out on the brain tissue using primers to VZV ORF68 and ORF62 genes and the cellular gene coding for GAPDH. (B) VZV genome copies. Using a standard curve, the number of genome copies was calculated from the PCR measurements in panel A. Given a known number of cells in the positive control, the number of genome copies was calculated. (C) VZV glycoprotein gene transcripts. Reverse transcription (RT)-qPCR measurements were made using the primers in Table 1 against cDNA from VZV-infected MRC-5 fibroblast cells (dark gray) or from brain tissue (light gray). (D) VZV regulatory gene transcripts. The herpes simplex ortholog is listed beneath each VZV gene.

In an earlier report, we had only amplified one VZV gene (ORF62) from brain tissue (8). To better assess the status of the viral genome within the CNS, we performed more complete DNA and RNA analyses. To this end, we extracted DNA and mRNA from subsections of the frozen tissue. We measured copies of both cellular and viral DNA using primers to VZV open reading frame 62 (ORF62) and ORF68 and the cellular gene coding for GAPDH (glyceraldehyde-3-phosphate dehydrogenase) in the brain tissue, along with both positive (DNA from VZV infected melanoma cells) and negative (DNA from water) controls (Fig. 1A). There was moderate amplification of viral DNA in the brain. Using a standard curve, the PCR data in panel A were converted to the number of VZV genome copies (Fig. 1B). Given a known estimate of the number of cells in the positive control, we computed the number of VZV genome copies per cell. Based on similar GAPDH amplifications in the positive control and the brain tissue in Fig. 1A, we reasoned that the brain tissue contained a similar number of cells to the diluted positive control. Thus, we predicted 3 to 7 copies per cell, compared to the positive control with 11,000 copies per cell. To test the latter possibility that DNA replication was restricted in the human brain, we carried out quantitative PCR (qPCR) measurements against cDNA from VZV-infected MRC-5 fibroblast cells (positive control) versus cDNA from the brain tissue using the primers listed in Table 1 to a subset of VZV glycoprotein and regulatory transcripts (Fig. 1C and D). The GAPDH threshold cycle (*C_T_*) was 8 cycles later for the brain tissue than the positive control, indicating 2^8^ fewer cells. Assuming there are 1 million cells in the positive control, then the small piece of brain tissue consisted of approximately 4,000 cells. In general, the level of VZV transcripts in the brain tissue was consistently less than that of the positive control by 2^4^ to 2^6^, a result suggesting that a productive infection was occurring in far fewer than 10% of the cells within the brain tissue.

**TABLE 1:** VZV primers used in this study

VZV gene	Primer sequence	
	Forward	Reverse
ORF4	5′-CTC GTA CTC TCC CCG AAC TG-3′	5′-CTA AAA CAC CGG CCA GAC AT-3′
ORF10	5′-ATT TTC CTG GCG TTT GTA CG-3′	5′-AGG CAG ACG CTG TTA GTG GT-3′
ORF14	5′-GGA ACT CGA CGG ACC TAT CA-3′	5′-CCC AAT GTC CCA AGG ATA GA-3′
ORF21	5′-GTT GGA CCC GAC TGG ATA AA-3′	5′-TTT AAA CGC CAT ACC CCA AA-3′
ORF29	5′-TCT TGT GAA CCA AGC CAT GA-3′	5′-CGT GTG GGC TTT AAT TGG AT-3′
ORF31	5′-CGT GGG ATT ATT GGT TTT GG-3′	5′-CGA CGG TTC AGT GTT TTG TG-3′
ORF37	5′-GCT CAT TCT TCC TCC AGC TGT CC-3′	5′-CAG TGC GAC CGT CTC TAT GA-3′
ORF60	5′-TCG GGA ATC GTG GTT AAG AC-3′	5′-TTT CCG GGA TAT TTG TGG AA-3′
ORF61	5′-CAA ACC TGG ACC TGG AAA GA-3′	5′-GAA ATA AAC GGC GCT TAG CA-3′
ORF62	5′-CCT TGG AAA CCA CAT GAT CGT-3′	5′-AGC AGA AGC CTC CTC GAC AA-3′
ORF63	5′-TGA AGA CGA TAG CGA CGA TG-3′	5′-TCC CCG TCT CGA TAA CAA TC-3′
ORF66	5′-GCA ACC TCC CCA TAT GTG AC-3′	5′-TTC CAG CCC AGC CAT AAT AC-3′
ORF67	5′-TCC ACG TTA CCC GAA AAG TC-3′	5′-GGA TTC TGG TGT CGC ATT TT-3′
ORF68	5′-CCC CGT AAA CCC CGG AAC GT-3′	5′-CCC GTG ACT CCC TCC AAT CGC-3′

### Survey of infected cells within focal VZV encephalitis.

**FIG 2:**
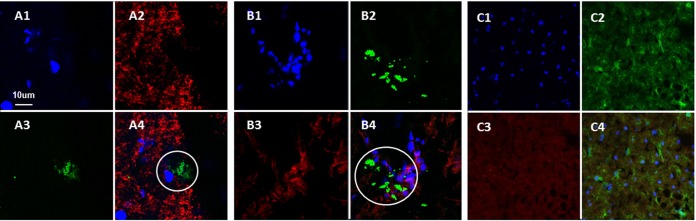
Lack of colocalization of VZV glycoproteins with neurofilaments, microglia, and oligodendrocytes. Three individual images and one merged image are included for each panel. Brain sections were labeled with rabbit antineurofilament antibody (red in panels A), IB4 lectin 568 conjugate for microglia (red in panels B), and murine RIP MAb for oligodendrocytes (red in panels C). Sections were also labeled with Hoechst H33342 (blue in panels A to C), anti-VZV gE MAb (green in panels A and B), and anti-VZV gH MAb (green in panels C). Circles indicate areas of detectable VZV antigen that do not colocalize with neurofilaments (A) or microglia (B).

After collecting the genomic data, we postulated that neurons would be the cell in which infection would be most easily detected. Therefore, we selected an established antibody probe for neurons and two anti-VZV monoclonal antibody probes. Based on the fact that VZV gE (ORF68) is an abundant gene product during VZV infection, we chose a well-characterized anti-gE MAb, 3B3, with high-affinity binding for a linear epitope that is known not to cross-react with uninfected brain tissue (22, 23). When we examined samples that were dually labeled for VZV gE and neuronal filament by confocal microscopy, we observed scattered foci of VZV gE reactivity, but virtually no VZV gE immunolabeling was found in neurons (Fig. 2A). We expanded this observation by obtaining a z-stack of images through the focus of gE immunolabel and confirmed that the gE immunolabel did not colocalize with cells displaying neuronal staining.

In order to investigate whether virus was present in microglia, we performed a similar confocal microscopy experiment after labeling cells with the Griffonia plant isolectin B4, which adheres to the cell wall of microglia. As seen in Fig. 2B, gE immunolabel was again detected but did not colocalize with individual microglial cells when a z-stack was examined. Thereafter, we investigated whether VZV infection was found in a third cell type, namely, oligodendrocytes. Because the anti-oligodendrocyte antibody was murine, we selected a human anti-gH (ORF37) monoclonal antibody as the VZV probe to facilitate labeling with two fluoroprobes of different wavelengths. This experiment produced a pivotal result—namely, VZV antigen was detected in stellate cells that did not coimmunolabel with the oligodendrocyte antibody (Fig. 2C). The absence of any RIP-positive cells indicated that this brain section was taken from gray matter. Because of the distinctive stellate morphology of the infected cells, we postulated that VZV infection may be more easily detectable in astrocytes.

### Infected astrocytes within focal VZV encephalitis.

**FIG 3:**
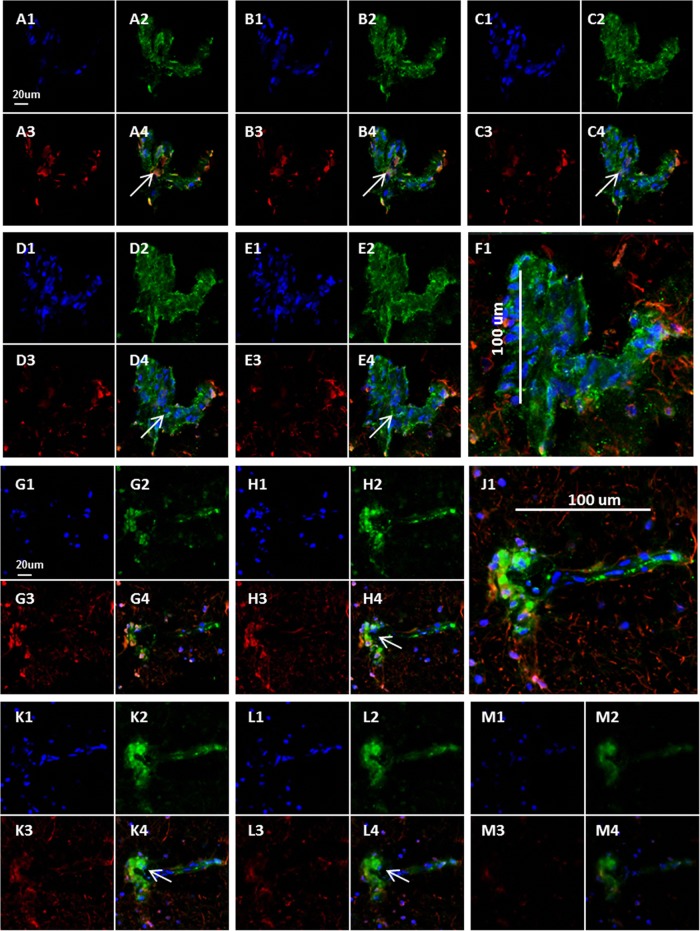
Colocalization of VZV glycoproteins with astrocytes. Twelve images from two z-series are shown. (A to F) Brain section from the first brain section labeled with Hoechst H33342 (blue in panels A1 to E1), human anti-VZV gH MAb (green in panels A2 to E2), and murine anti-GFAP antibody (red in panels A3 to E3). The merge of panels F1 to -4 is shown in panel F1, with a scale bar to outline the astrocyte. (G to M) Brain section from second brain section labeled with Hoechst H33342 (blue in panels G1, H1, K1, L1, and M1), human anti-VZV gH MAb (green in panels G2, H2, K2, L2, and M2), and murine anti-GFAP antibody (red in panels G3, H3, K3, L3, and M3). The merge of panels J1 to -4 is shown in panel J1, with a scale bar to outline the astrocyte. Colocalization was seen (arrows).

Immunodetection of glial fibrillary acidic protein (GFAP) is the standard method for identifying intermediate filaments in activated astrocytes (24). Because the anti-astrocyte antibodies were of murine origin, we continued to use the human anti-gH MAb as the VZV probe for this set of experiments. As noted in Materials and Methods, we selected two anti-GFAP reagents, one of which was conjugated with a Cy3 fluoroprobe. During these confocal microscopy experiments, it became readily apparent that there was considerable colocalization of the VZV antigen within cells immunolabeled with GFAP reagent. Two representative z-stacks are shown in Fig. 3. Furthermore, several of the immunopositive cells were large. After further examination, we concluded that some large GFAP-positive VZV-infected cells were clustered into aggregates. Since gH is one component of the VZV fusion complex, we reasoned that gH would clearly define syncytia (25, 26).

**FIG 4:**
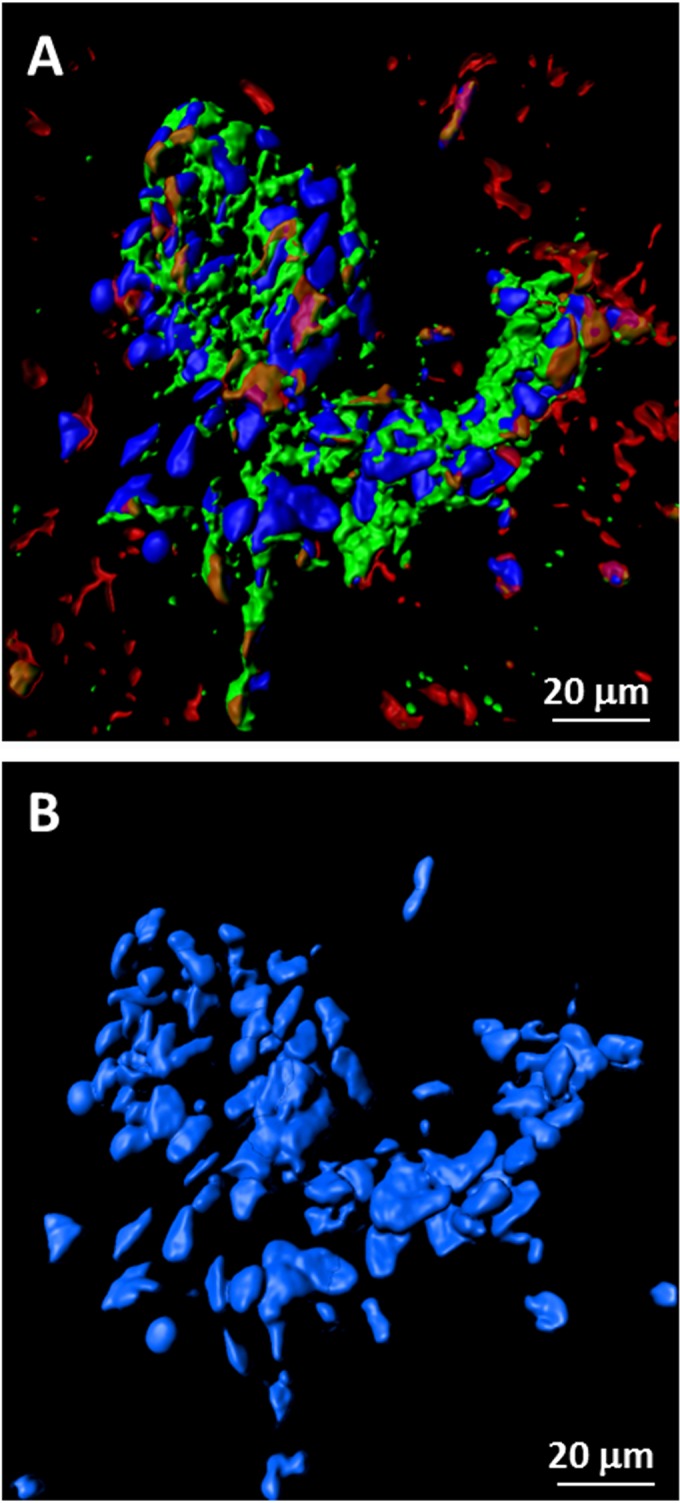
Syncytium of VZV-infected astrocytes. Confocal images from Fig. 3 were converted by Imaris software into 3D animations (18). One frame from an animation was selected to illustrate the large number of nuclei (∼80) present within an area of fusion. Panel A retains all fluorescent labels; panel B includes only the blue double-stranded DNA (dsDNA) stain in order to delineate nuclei.

To further assess the aggregates of infected cells, z-stacks containing 40 slices were converted by Imaris software into 3D animations; the 3D images confirmed that extensive VZV gH-positive antigen was found within GFAP-positive cells. A 2D frame from one of the animations shows a syncytium of astrocytes containing about 80 nuclei (Fig. 4). Because of the importance of this set of immunolabeling experiments, over 200 confocal micrographs were examined. During the same investigations, we failed to find evidence of VZV gH reactivity within either circular structures (2D images) or cylindrical structures (3D animations) that could represent blood vessels.

### Further analysis of aggregates.

**FIG 5:**
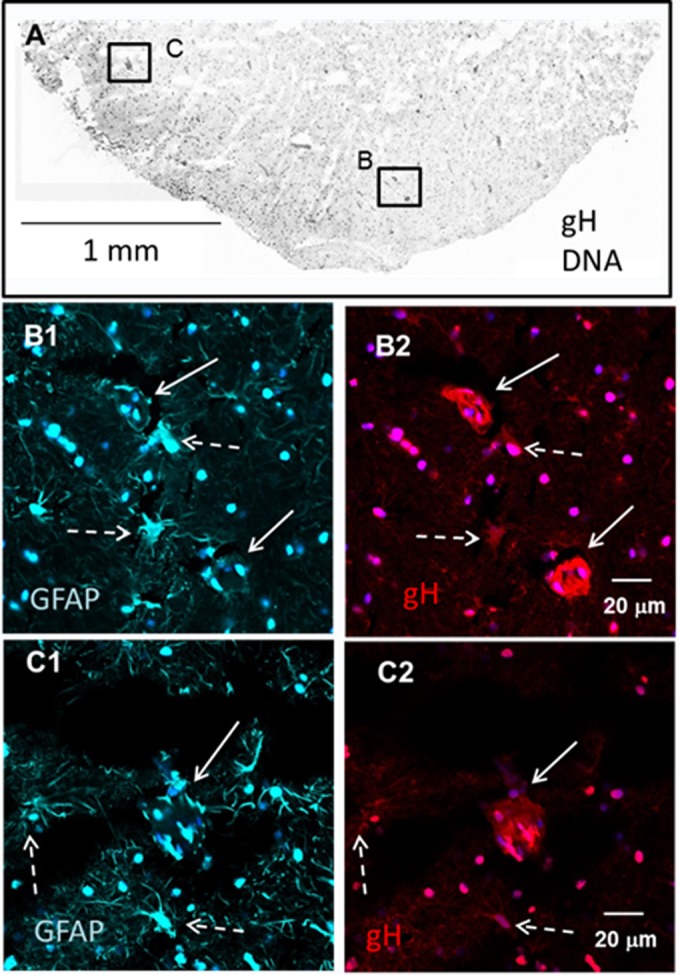
GFAP-positive cellular aggregates in the human brain. Sections were cut from the frozen brain tissue and immunolabeled with monoclonal antibodies to VZV gH and GFAP. (A) Landscape view. Many images were converted to black and white and combined to make a montage of VZV gH and nuclear staining (Hoechst H33342). Dense scattered areas of staining are readily apparent. Two dense areas are marked by boxes B and C. (B1 and -2). Fluorescent images of GFAP (B1) and gH (B2) positivity in box B within panel A. Two areas (solid white arrows) are VZV gH positive but show a downregulation of GFAP. Neighboring astrocytes (dashed white arrows) showed early infection and upregulation of GFAP. (C1 and -2). Fluorescent images of GFAP (C1) and gH (C2) positivity in box C within panel A. A single large infected aggregate (solid white arrow) also exhibited a downregulation of GFAP, while neighboring astrocytes (dashed white arrows) showed early infection and upregulation of GFAP.

In order to further clarify the relationship of GPAP immunolabeling to these aggregates, we probed a larger section of the flash-frozen brain tissue with antibodies to VZV gH and the astrocytic marker GFAP and visualized at low magnification (Fig. 5). A wide-area montage revealed a moderate number of dense gH-positive cellular aggregates that were scattered across the brain section (Fig. 5A). Closer examination of two gH-positive areas (Fig. 5B and C) displayed a downregulation of GFAP immunolabel within the aggregates, while neighboring astrocytes showed signs of early infection and upregulation of GFAP. These results strongly supported previous observations that VZV infection of astrocytes caused a downregulation of GFAP expression (27).

### Detection of the VZV true late gC protein in gH-positive cells.

**FIG 6:**
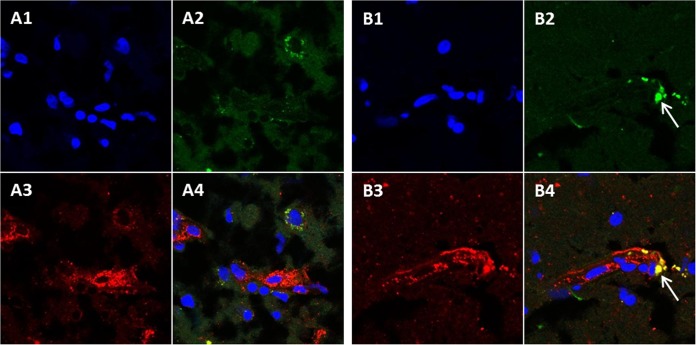
VZV glycoproteins gC and gH in the brain. Brain section labeled with Hoechst H33342 (blue in panels A1 and B1), murine anti-VZV gC MAb (green in panels A2 and B2), and human anti-VZV gH MAb (red in panels A3 and B3). The merge of panels A1 to A3 and B1 to B3 is shown in panels A4 and B4. The white arrows in panels B2 and B4 indicate areas of gC and gH colocalization.

To determine whether VZV can complete its replication cycle in astrocytes, we next investigated whether the true late (gamma-2) gC protein (ORF14) was detectable (15). As a positive control for infected astrocytes (Fig. 3), we again selected the anti-gH antibody of human origin. The anti-gC antibody is of murine origin. As can be seen in Fig. 6, VZV gC was detected within infected cells, but the relative amount of gC immunolabeling was considerably less than with gH immunolabeling. The limited gC production combined with near-normal gC transcription results (Fig. 1) suggested a delayed expression of the gamma-2 viral products in astrocytes, similar to that seen previously in cultured neurons (28, 29).

### Cytoarchitecture of the VZV-infected human brain.

**FIG 7:**
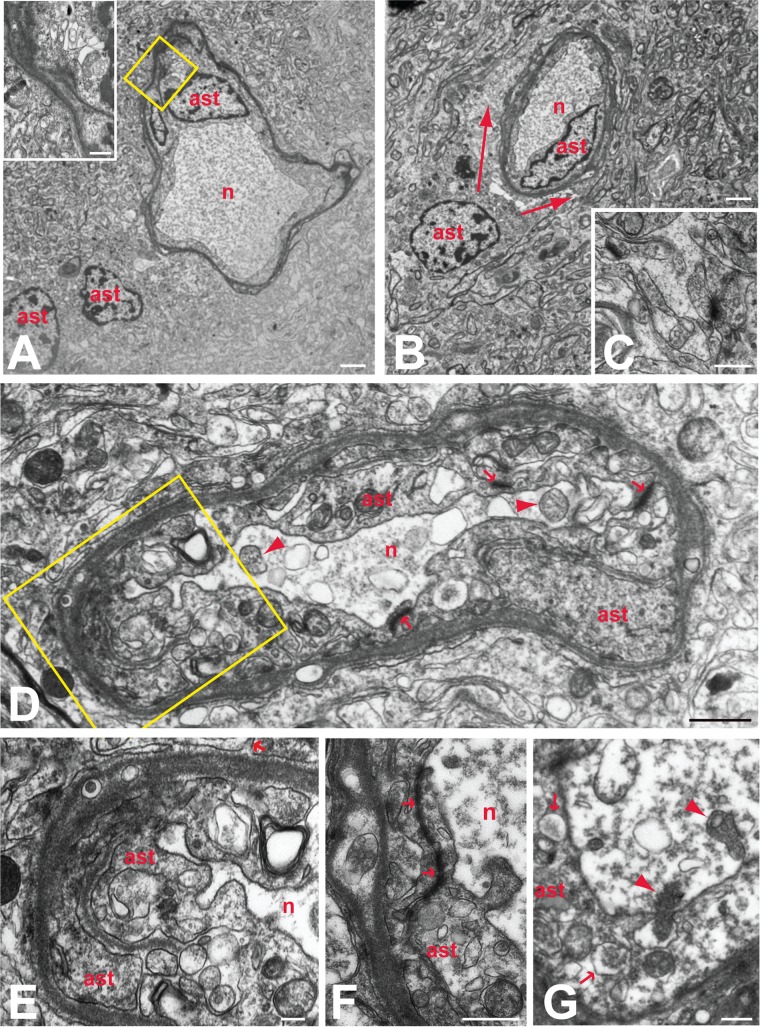
Reactive astrocytes and astrogliosis in VZV-infected human brain. The relationships of reactive astroglia (ast) to degenerating neurons (n) are illustrated in the electron micrographs. Barrier astroctyes formed intimate relations with degenerating neurons, entirely isolating the degenerating neurons from the surrounding neuropil (A to D). In some cases, more than one reactive astrocyte contributed to this barrier (A), with the cells fusing to form a syncytium (inset in panel A). These astrocytes were also the source of thin interleaving processes that isolated synaptic profiles within the neuropil (C; arrow in panel E). Reactive astroglia in the adjacent neuropil also contributed processes that surrounded barrier astrocytes (defined by arrows in panel B), further isolating degenerating neurons from the brain parenchyma. Prominent accumulation of dense proteinaceous material underlying the outer plasma membranes was a characteristic feature of all barrier astrocytes (A, B, D to F). This material also was present in processes that invaded deeper layers of the astrocyte barrier to fuse with other processes (boxed area in panel D, which is shown at higher magnification in panel E). Tight junctions formed between processes within the glial barrier (arrows in panels D and F) contributed to the complete isolation of ensconced neurons. Neurons isolated by the barrier astrocytes characteristically contained accumulations of flocculent material, vacuolization, and mitochondria in various states of degeneration (arrowheads in panels D and G). Phagocytosis of degenerating neurons by barrier astrocytes was also a characteristic feature of barrier astrocytes. One of two degenerating mitochondria marked by arrowheads in panel G is within a portion of the soma being phagocytosed by the barrier astrocyte. Arrowheads within that barrier astrocyte identify phagosomes that are similar in morphology to the cytoplasm within the portion of the neuron being phagocytosed. Marker bars by panel: A and B, 2 μm; C and G, 500 nm; D, 1 μm; E, 200 nm; F, 600 nm.

To gain further insight into the role and extent of reactive gliosis in VZV-induced neuropathology, we examined a portion of the excised temporal lobe tissue using transmission electron microscopy (TEM). Observers at both the University of Iowa and the University of Pittsburgh independently examined ultrathin sections of the tissue and identified the cell types (30). Systematic scanning of numerous sections revealed focal aggregations of degenerating neurons within the tissue. In every case, one or more astrocytes surrounded the degenerating neurons, entirely isolating them from the adjacent brain parenchyma (Fig. 7A, B, and D). Tight junctions enhanced the integrity of this barrier by binding the complex interlacing network of astrocytic processes surrounding each neuron. A remarkable uniformity in morphology characterized each of these glial barriers. Particularly notable in this regard was accumulation of electron-dense proteinaceous material at the outermost border of the barrier, giving the appearance of a basal lamina surrounding the cell (Fig. 7A, B, and D to F). However, careful analysis at high magnification demonstrated that the proteinaceous material was within the limiting membrane of the astrocyte, forming a continuous band adjacent to the plasmalemma of the outermost processes. High-magnification analysis also demonstrated that the dense material was continuous into branches of the outer process that invaded deeper layers and fused with processes exhibiting a more lucent cytoplasm. The fusion of processes within this complicated interleaving network presented clear evidence of syncytium formation that was consistent with confocal microscopic observations (Fig. 4). Evidence of syncytium formation was also commonly observed by the continuity of cytoplasm between profiles with separate nuclei (inset in Fig. 7A).

Examination of the interface between degenerating neurons and the barrier astrocytes revealed clear evidence of phagocytic activity (Fig. 7G). Astrocyte processes routinely invaded the soma of degenerating neurons, and phagosomes were prevalent throughout the astrocyte cytoplasm. Thus, it was evident that the barrier astrocytes not only isolated degenerating neurons from the brain parenchyma but also were involved in actively removing them through phagocytosis. Systematic analysis of numerous reactive astrocytes revealed a paucity of viral particles. Occasional evidence of capsid formation was observed in the nuclei of the barrier astrocytes intimately associated with degenerating neurons. However, enveloped virions were underrepresented within the cytoplasm of these cells. Reactive astroglia in the surrounding brain parenchyma rarely exhibited evidence of viral replication.

### Cytoarchitecture of rodent brain infected with pseudorabies virus.

**FIG 8:**
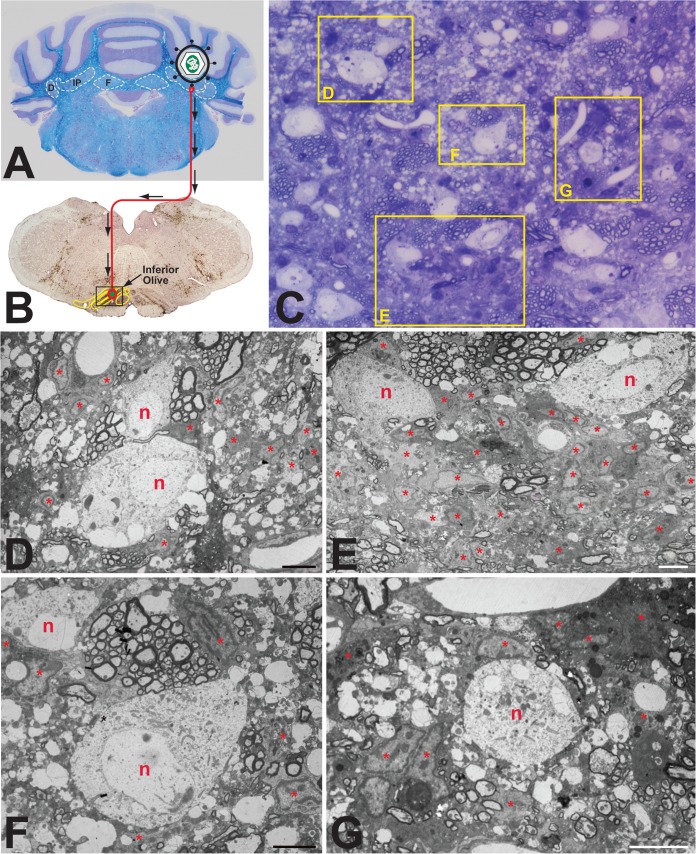
Experimental paradigm and cellular response to PRV infection in rat brain. (A and B) Paradigm. Injection of the PRV Bartha strain into the interpositus nucleus of the rat cerebellum (A) resulted in retrograde spread of virus to infect neurons within the inferior olive (B). The brown reaction product visible in panel B represents immunocytochemical localization of viral antigens marking infected cells within the inferior olive and surrounding brain stem 2 days after injection of virus. (C) Thick section. The panel is a toluidine blue-stained 1-μm section from the boxed area shown in panel B. Intensely basophilic cells define reactive glia in relation to infected neurons. (D to G) Thin sections. Processing of tissue adjacent to the thick section for TEM analysis revealed infected neurons (n) and reactive astrogliosis (ast) within the cerebellum. Because the number of astrocytes was so large, some are labeled internally with an asterisk. Several infected neurons exhibited vacuolization and were intimately associated with reactive glial cells and their processes. Marker bars for panels D to G are 6 μm.

**FIG 9:**
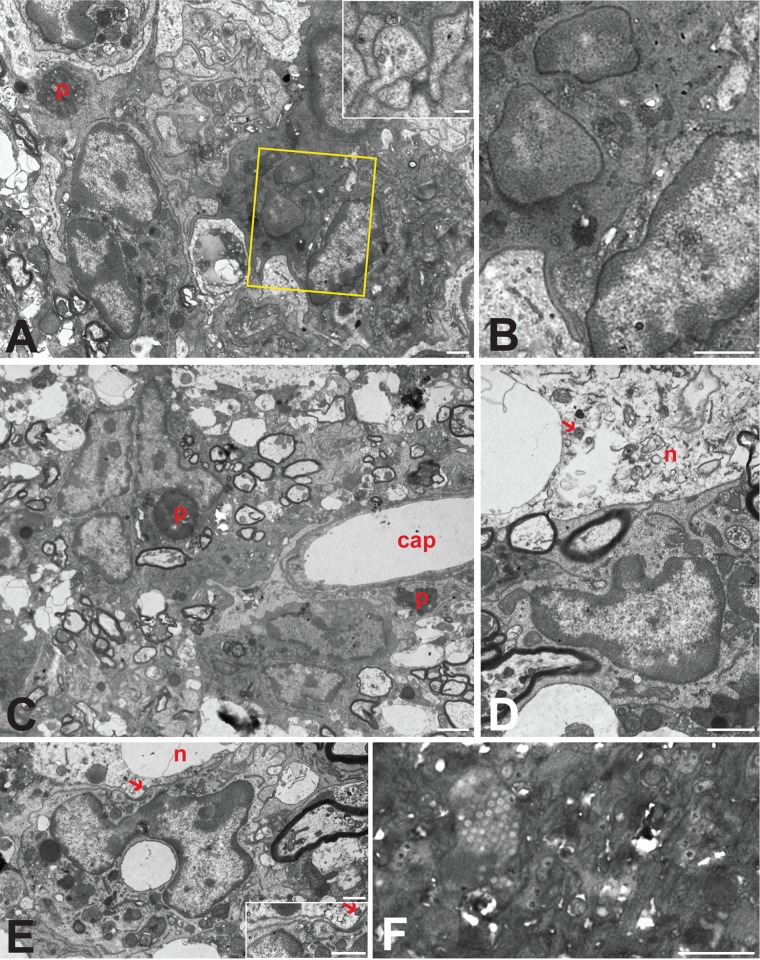
Astrogliosis in the PRV-infected rat brain. The interrelations of multicellular aggregates of reactive astroglia adjacent to infected neurons from the fields shown in Fig. 8 are illustrated at higher magnification. Infected neurons exhibited pathological vacuolization (D and E) and contained mature virions (arrows). Aggregates of reactive glia in the immediate vicinity were directly apposed to infected neurons (D and E). Astroglia in aggregates also gave rise to interdigitating processes that isolated infected neurons from brain parenchyma and often contained capsids with no sign of envelopment (inset in panel A). Cells that contained larger number of capsids in either the nucleus (F) or cytoplasm (boxed portions are of panel A shown at higher magnification in panel B) typically exhibited cytoplasm that was more electron dense and also commonly displayed evidence of syncytium formation (B). Phagosomes (p) provided evidence of phagocytic activity by reactive astroglia within each aggregate, and astrocytes within the aggregate also completely invested adjacent capillaries (cap in panel C). Marker bars by panel: A, B, D, E, and F and inset in E, 1 μm; inset in A, 200 nm; C, 2 μm.

Because the reactive astrogliosis observed in the samples of VZV-infected human brain tissue exhibited features similar to those previously documented in the rat CNS following PRV infection (31), we conducted a new comparative analysis of this response in rats infected with PRV strain RV-152 (Fig. 8). This PRV recombinant of the Bartha strain is transported retrogradely through neural circuits and produces cytopathology at advanced stages of viral replication. In this experiment, the virus was injected into cerebellar neurons that receive projections from neurons in the inferior olive (Fig. 8A and B). The glial response to viral replication was then characterized 48 h later. Examination of toluidine blue-stained thick sections revealed aggregations of intensely basophilic glial cells adjacent to infected neurons (Fig. 8C). Electron microscopic examination of adjacent ultrathin sections demonstrated that these multicellular aggregates were intimately associated with infected neurons, often investing the neuron with a dense array of cells and processes (Fig. 8D to G). Analysis of these glial aggregates revealed that they contained a dense concentration of astrocytes and microglia, consistent with earlier reports of the nonneuronal response to PRV infection (31, 32). The reactive astrogliosis displayed morphological features similar to those observed in the VZV-infected human brain tissue. These shared features included the formation of complex interleaving process networks (Fig. 9A), evidence of syncytium formation (Fig. 9B and C), intimate associations of astrocytes with somata of infected neurons (Fig. 9D and E), and the presence of phagosomes within the cellular cytoplasm (Fig. 9A and C). A subset of reactive astrocytes also contained capsids within the nucleus and cytoplasm (Fig. 9A to F), but enveloped pseudorabies virions at the plasma membrane were difficult to find.

## DISCUSSION

A central finding of this report is that astrocytes are an important component of the CNS response to VZV neurotropism in humans who recover from their infection. The role of astrocytes during the CNS stress response is a subject of increasing investigation (33, 34). Astrocytes outnumber neurons in the human brain. While there are an estimated 16 billion neurons in the cerebral cortex, the number of astrocytes is presumably higher (35). Astrocytes in humans are larger and more complex that those in most primates and all other mammals. Furthermore, the 3D territory over which a human astrocyte extends is much greater than that seen in all nonhuman astrocytes. For example, the processes extending from a single human cortical astrocyte are estimated to encompass a domain including hundreds, perhaps thousands, of neuronal synapses (36).

Astrocytes in the human cortex are classified into 4 subgroups: (i) interlaminar, (ii) protoplasmic, (iii) varicose projection, and (iv) fibrous (37). The first three are located entirely or in part within the gray matter, while the fibrous astrocytes are found mainly in white matter. The extended family includes Muller glia in the retina and Bergmann glia in the cerebellum. Astrocytes are essential polyvalent cells in the brain. They are no longer considered just the glue (“glia” is derived from the Latin word for glue) or the matrix that holds the rest of the brain together (38). Instead, they participate in several functions, including energy metabolism, ion exchange, and glutamate homeostasis, as well as acting as sentinels for viral infection via their expression of the Toll-like receptor 3 (TLR3) (39, 40). Furthermore, astrocytes are involved in cross talk with both neurons and microglia; fiber-like processes of astrocytes are in contact with both synapses and blood vessels. Through these shared connections, astrocytic end feet titrate blood flow in response to various levels of synaptic activity.

Gliosis is the term that describes a proliferation of glial cells in the CNS as a response to injury (34). Gliosis has not been recognized widely as a consequence of VZV infection in the CNS, although gliosis was observed by standard hematoxylin and eosin (H&E) visualization in this brain tissue (8). As noted in the initial report, the pathology was mainly concentrated in the gray matter of the temporal lobe. Furthermore, our data convincingly demonstrated that the predominant infected cell was the astrocyte. Based on their size and morphology, we postulate that they were mostly protoplasmic astrocytes. These are the most numerous of the 4 subclasses, and they are located in this region of the temporal lobe. Their diameters range from <100 to >400 μm, with an average around 150 μm. Aggregates of fused astrocytes that immunolabeled with the VZV fusion complex anti-gH antibody were also observed.

There is also a question of whether astrocytes were infected primarily or only secondarily after infection of a neuron. Obviously we did not have an earlier brain biopsy specimen to examine. Furthermore, there is no good animal model for VZV encephalitis. To answer that important question, therefore, we examined earlier studies of the spatiotemporal responses of neuronal and glial cells to pseudorabies (PRV) infection in the CNS (31, 32). PRV is a related animal alphaherpesvirus in the same Varicellovirus genus as VZV; neither VZV nor PRV harbors the herpes simplex virus 1 (HSV-1) neurovirulence protein (41). In an earlier PRV study, rats were infected in the stomach, after which the progressive course of viral pathology was examined at several time points in the vagus nerve and finally in the amygdala within the mesial temporal lobes. In the amygdala, infection of GFAP-positive astrocytes was noted surrounding infected neurons. Furthermore, PRV infection in astrocytes was considered to be abortive, in that relatively few completely enveloped virions were observed by TEM. Thus, there was little or no spread of infection beyond the astrocytes that were initially infected (32). For this current project, a second independent investigation of a PRV-infected brain was carried out. Astrocytes were easily identified around necrotic neurons; thus, the second study confirmed and expanded the observations about astrocytes seen in the original study. Taken together, our results with VZV infection of astrocytes in the human brain appear very similar to PRV infection within astrocytes in the rat brain.

With regard to VZV infection in the brain, another question is why the antiviral property of astrocytes was not previously recognized. One explanation is that VZV meningoencephalitis and vasculopathy can occur in the absence of skin rash and would be difficult to diagnose (11). A second explanation is that nearly all prior VZV investigations in the brain were performed on specimens collected at autopsy at various times after death. We postulate that humans with fatal VZV CNS infections likely represent situations in which (i) the human host was immunocompromised and (ii) the astrocytic defensive wall failed. As an example, there was a case report of fatal CNS disease due to disseminated VZV infection in an AIDS patient before current effective therapeutic protocols (42). The authors recognized that astrocytes were infected as part of the pathological process in the brain, but they did not postulate a defensive role. The same group subsequently studied VZV replication in astrocyte cell lines and showed the VZV replication proceeded more quickly in astrocytes than in neurons but more slowly than in human fibroblasts (27, 43). The latter situations suggest that cross talk between astrocytes and neurons is required for astrocytes to form a defensive perimeter in the infected human brain. In a similar manner to protection afforded by astrogliosis following other traumatic brain events, therefore, astrogliosis appears to be a hallmark of a successful innate and adaptive immune response after VZV reactivation and transit into the brain (33, 34).

Finally, the case in this report does not represent an example of VZV vasculopathy as a cause of the CNS neuropathology (44, 45). However, we suggest that the underlying neuropathogenesis is related. Namely, by chance, this human brain sample was infected by VZV exiting a branch of the trigeminal nerve that innervates the outer gray matter of the lateral temporal lobe, not a region with larger blood vessels. A greater number of trigeminal afferent fibers travel along the carotid arteries within the white matter and therefore can convey reactive virus from the ganglion to the adventitia of arteries, eventually leading to inflammation and stroke (46, 47).
